# Effect of Tunicamycin on Viability, Motility, Reactive Oxygen Species, Nitric Oxide, and Lipid Peroxidation in Boar Sperm

**DOI:** 10.3390/ani15101422

**Published:** 2025-05-14

**Authors:** Seunghyung Lee

**Affiliations:** College of Animal Life Sciences, Kangwon National University, Chuncheon 24341, Republic of Korea; s.lee@kangwon.ac.kr; Tel.: +82-33-250-8637

**Keywords:** tunicamycin, reactive oxygen species, nitric oxide, lipid peroxidation, boar sperm

## Abstract

Tunicamycin induces endoplasmic reticulum stress in mammalian cells. Our study aimed to investigate the effect of tunicamycin on the motility and viability of sperm, reactive oxygen species, nitric oxide, and lipid peroxidation in boar sperm. We treated 1.0, 2.0, 5.0, and 10 μM of tunicamycin in boar semen, and experimental treatments were performed. The viability (55.44%, 53.20, and 40.00%, *p* < 0.05) and motility (73.28%, 71.48%, and 54.48%, *p* < 0.05) of sperm at 2.0, 5.0, and 10.0 μM were decreased by tunicamycin, and the levels of reactive oxygen species and lipid peroxidation in tunicamycin-treated boar semen were increased (*p* < 0.05). However, the nitric oxide level was not changed by tunicamycin. Based on the results, we indicated that tunicamycin induces cell death by increasing oxidative stress in boar sperm, which may be the cause of decreased sperm viability and motility. Thus, we suggest that tunicamycin may induce cell death due to oxidative stress by reactive oxygen species and lipid peroxidation.

## Simple Summary

Tunicamycin regulates endoplasmic reticulum stress and oxidative stress. Sperm motility and viability are damaged by reactive oxygen species. We investigated the effect of tunicamycin on sperm viability, motility, reactive oxygen species, nitric oxide, and lipid peroxidation in boar sperm. As a result, tunicamycin inhibited sperm viability and motility, and elevated the reactive oxygen species and lipid peroxidation levels. Therefore, we suggested that tunicamycin may regulate oxidative stress by reactive oxygen species and lipid peroxidation in boar semen.

## 1. Introduction

Tunicamycin specifically targets the early stages of N-linked glycosylation, a vital process for glycoprotein synthesis [1]. By inhibiting this pathway, tunicamycin induces endoplasmic reticulum stress, leading to the activation of the unfolded protein response (UPR). Stress activates UPR pathways, which can initiate apoptotic signaling [2]. The stress response can result in apoptosis if the cellular is irreparable. The effects of tunicamycin on various cell types have been extensively studied, revealing both apoptotic and non-apoptotic results. In hepatocellular carcinoma (HCC) cells, tunicamycin increased pro-apoptotic proteins and decreased anti-apoptotic proteins. Also, tunicamycin suppressed migration and invasion by downregulating CD44 standard form (CD44s) expression and affecting the extracellular signal-regulated kinase 1/2 (ERK1/2) signaling pathway, which are critical for metastatic potential [3,4]. Endoplasmic reticulum stress induced by tunicamycin can trigger autophagy, a cellular degradation process. The interplay between autophagy and apoptosis under tunicamycin treatment is complex and context-dependent, influencing cell survival or death. Also, the impact of tunicamycin varies among different cell types. Some studies reported tunicamycin-induced endoplasmic reticulum stress in neuronal, cancer, endothelial, and immune cells, but research on sperm in humans and animals is limited.

Tunicamycin induces oxidative stress, characterized by an imbalance between reactive oxygen species production and the antioxidant defenses in the cells [5]. Tunicamycin induces reactive oxygen species production, which can cause cellular damage and activate apoptotic pathways. This reactive oxygen species generation contributes to the downregulation of survival proteins like survivin, promoting cell death in leukemia cells [6]. Tunicamycin treatment disrupts sulfur amino acid metabolism, leading to decreased hepatic glutathione levels. Glutathione is a critical antioxidant, and its reduction impairs the liver’s ability to neutralize reactive oxygen species, resulting in increased oxidative stress [7]. The accumulation of reactive oxygen species leads to mitochondrial membrane potential dissipation and activation of apoptotic pathways. Antioxidants like N-acetyl cysteine (NAC) can mitigate these effects, highlighting the role of reactive oxygen species in tunicamycin-induced cytotoxicity [8]. Tunicamycin exposure enhances reactive oxygen species production in chondrocytes, leading to increased expression of oxidative stress markers such as heme oxygenase and ferritin heavy chains [9]. This upregulation contributes to cellular damage and dysfunction. Increased reactive oxygen species levels result in lipid peroxidation, producing reactive aldehydes like malondialdehyde. Elevated malondialdehyde levels are indicative of oxidative damage and are observed in tunicamycin-treated cells [10]. Also, tunicamycin induces oxidative stress in endothelial cells by reducing malondialdehyde levels [11]. This suggests that tunicamycin-induced endoplasmic reticulum stress can lead to oxidative damage in vascular tissues, which may be mitigated by certain antioxidants. Also, oxidative stress regulates reactive oxygen species, nitric oxide, and lipid peroxidation in mammalian cells. However, the role of tunicamycin in boar sperm is unknown.

Studies on the effects of tunicamycin on sperm cells in boars are insufficient. There is no study on the relationship between tunicamycin and oxidative stress (reactive oxygen species, nitric oxide, and lipid peroxidation) in boar sperm. Therefore, this study was conducted to investigate the effect of tunicamycin on boar sperm cells. We examined the effects of tunicamycin on sperm motility, viability, reactive oxygen species, nitric oxide, and lipid peroxidation in boar sperm.

## 2. Materials and Methods

### 2.1. Chemicals

Tunicamycin was purchased from Sigma-Aldrich (St. Louis, MO, USA). Unless otherwise indicated, all reagents used in this study were purchased from Sigma-Aldrich. All experiments were performed at room temperature in a clean room in our laboratory (Kangwon National University, Chuncheon, Republic of Korea). The sperm samples were incubated with or without 1, 2, 5, and 10 μM tunicamycin. We designed the experiment as follows: sperm motility and viability, reactive oxygen species and nitric oxide production, and lipid peroxidation determination.

### 2.2. Semen Preparation

We collected semen from ten crossbred pigs (Duroc × Yorkshire × Landrace, average ages = 28.7 ± 3.2; the artificial insemination center, Wonju, Republic of Korea). Boars were housed in 0.5 m^2^ slatted pens and fed a concentrated feed with free access to water. First, the semen was collected by a glove-hand method and diluted in a Modena extender (25.0 g/L glucose, 2.25 g/L ethylenediaminetetraacetic acid, 6.90 g/L sodium citrate, 1.00 g/L sodium bicarbonate, 5.65 g/L tris, 2.00 g/L citrate, 0.05 g/L cysteine, 0.30 g/L gentamicin sulfate, and pH 7.0). Then, the diluted semen was transported to the laboratory within an hour. Finally, the samples were resuspended in a Modena extender with tunicamycin to a concentration of 1.0 × 10^7^ sperm/mL. We used semen samples for motility, viability, reactive oxygen species, nitric oxide, and lipid peroxidation analysis experiments (3 times replicates from 10 semen samples in each pig). All experiments and guidelines were approved by the Institutional Animal Care and Use Committee of the University (KIACUC-09-0139, Kangwon National University, Chuncheon, Republic of Korea).

### 2.3. Sperm Motility

Sperm motility was subjectively evaluated using a standard method [12]. To measure the motility of treated sperm, a 10.0 μL sperm sample was placed on a pre-warmed slide glass, covered with a cover glass, and then placed in a warm chamber. The total sperm motility was subjectively assessed by visual estimations. Activated and moved sperm were counted. In at least five fields, sperm motility was determined and examined at a magnification of ×200 under a light microscope (Olympus, Tokyo, Japan).

### 2.4. Sperm Viability

Sperm viability was evaluated using a sperm Live/Dead kit (L-7011, Invitrogen, New York, NY, USA) according to a previous study [13]. The sperm samples were diluted with 40 nM SYBR-14 and 2.0 μM propidium iodide (PI), which were incubated at 38 °C for 5 min in a dark room, and then these samples were centrifuged at 410× *g* for 5 min. After the supernatant was carefully removed, the sample was re-suspended in Phosphate Buffered Saline (PBS). Then, sperm were measured using flow cytometry (488 nm, FACSCaliber, BD Biosciences, Franklin Lakes, NJ, USA). The data were analyzed using CELLQuest software (Version 6.0, Becton Dickinson, San Jose, CA, USA).

### 2.5. Reactive Oxygen Species

In the sperm, we determined the production of reactive oxygen species using 2′,7′-dichloro-fluorescein diacetate (DCF-DA, ROS assay kit, Invitrogen, New York, NY, USA) [14]. The DCF standard curve was used to quantify the produced ROS. Briefly, after centrifuging a 1.0 mL semen sample with a concentration of 1.0 × 10^7^ sperm/mL at 410× *g* for 5 min, 200.0 μL of the supernatant was mixed with 20.0 μL of 20.0 mM DCF-DA and incubated for 30 min in an incubator at 37 °C. Samples were centrifuged at 400× *g* for 5 min again. Finally, the sample was resuspended in PBS. Then, we measured the reactive oxygen species using flow cytometry (BD Biosciences).

### 2.6. Nitric Oxide

The production of nitric oxide was detected using a nitric oxide 4,5-diaminofluorescein diacetate (DAF-2 DA) reagent (Sigma, St. Louis, MO, USA). Nitric oxide in the boar semen was assessed as described previously [15]. Briefly, we used a concentration of 1.0 × 10^7^ sperm/mL in PBS at 410 g for washing for 10 min, and then DAF-2 DA was added to the suspension for a final concentration of 10.0 μM. Finally, the sample was incubated at 37 °C for an hour in a dark room. These samples were centrifuged at 400× *g* for 5 min. After that, we measured the incubated samples for NO production of sperm using flow cytometry.

### 2.7. Lipid Peroxidation

Lipid peroxidation of sperm was measured by the level of malondialdehyde through the reaction of thiobarbituric acid [16]. The concentration of the processed semen was diluted to 2 × 10^7^ sperm/mL using D-phosphate buffered saline, and 10.0 μL of 1.0 mM ferrous sulfate and 5.0 mM sodium ascorbate were added to 1 mL of sperm suspension and incubated for 1 hr under conditions of 37 °C and 5% CO_2_. After incubation, 250.0 μL of 40% trichloroacetic acid was added to the sperm suspension, and the suspension was incubated at 0 °C for 10 min, and then centrifuged at 2500× *g* for 10 min. After that, 1 mL of the supernatant was collected, 250.0 μL of 1% trichloroacetic acid was added, and the suspension was boiled at 100 °C for 10 min, cooled to room temperature, and the malondialdehyde value was measured. Malondialdehyde is the value measured by absorbance at a wavelength of 532 nm using a spectrophotometer (Amersham Biosciences, Piscataway, NJ, USA).

### 2.8. Statistical Analysis

One-way ANOVA, followed by the Fisher protected least significant difference (PLSD) analysis, was used for all statistical data analysis using StatView (SAS Institute, Cary, NC, USA). Data were presented as mean ± standard error. A *p*-value < 0.05 was considered significant.

## 3. Results

### 3.1. Effect of Tunicamycin on Sperm Motility and Viability in Boar Semen

The effect of tunicamycin on sperm motility and viability is shown in Table 1. Sperm motility in the 2.0 μM, 5.0 μM, and 10.0 μM tunicamycin-treated semen (73.28%, 71.48%, and 54.48%, respectively) was significantly decreased compared with 0.0 μM and 1.0 μM tunicamycin-treated semen (*p* < 0.05). However, the sperm motility was not significantly different between the non-treated group (79.68%) and the 1.0 μM tunicamycin-treated group (79.82%). The IC_50_ value of sperm motility was 6.37 μM.

We also evaluated the viability of sperm at tunicamycin concentrations in boar semen. Table 1 shows that sperm viability in tunicamycin-treated semen was significantly decreased in a dose-dependent manner, with 2.0 μM, 5.0 μM, and 10.0 μM tunicamycin (*p* < 0.05). The IC_50_ value of sperm viability was 5.76 μM. Therefore, we continually used concentrations of 1.0, 2.0, and 5.0 μM tunicamycin to determine the levels of reactive oxygen species, nitric oxide, and lipid peroxidation of sperm in boar semen.

### 3.2. Effect of Tunicamycin on Reactive Oxygen Species, Nitric Oxide, and Lipid Peroxidation in Boar Semen

**Figure 1:**
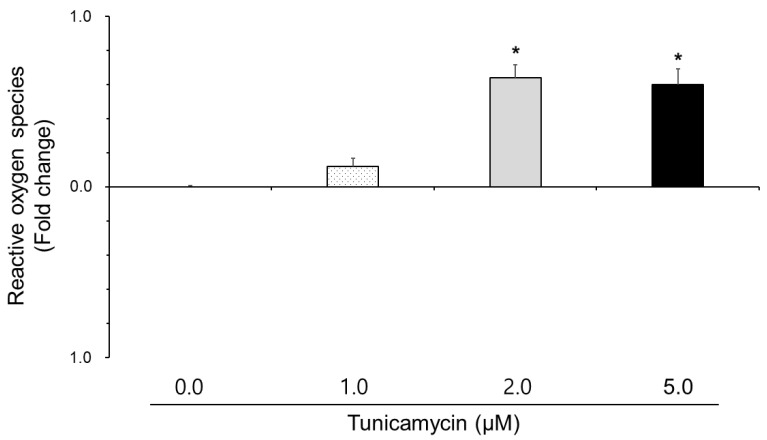
Effect of tunicamycin on reactive oxygen species in boar semen. Sperm were treated with 0.0, 1.0, 2.0, and 5.0 μM tunicamycin. Bars represent means ± standard error. Asterisks indicate significant differences (*, *p* < 0.05).

**Figure 2:**
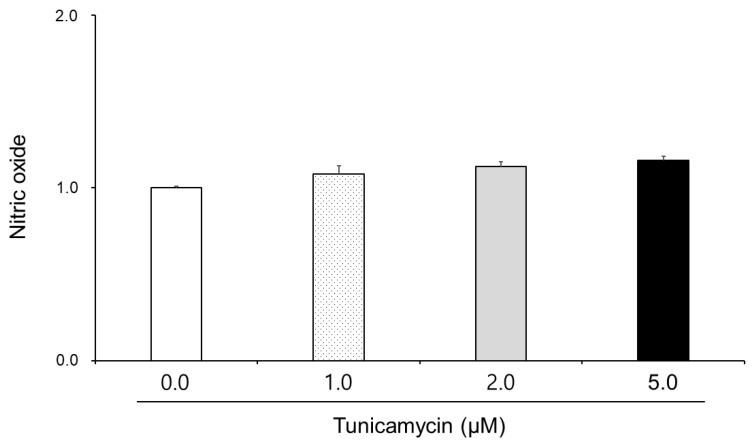
Effect of tunicamycin on nitric oxide in boar semen. Sperm were treated with 0.0, 1.0, 2.0, and 5.0 μM tunicamycin. Bars represent means ± standard error.

**Figure 3:**
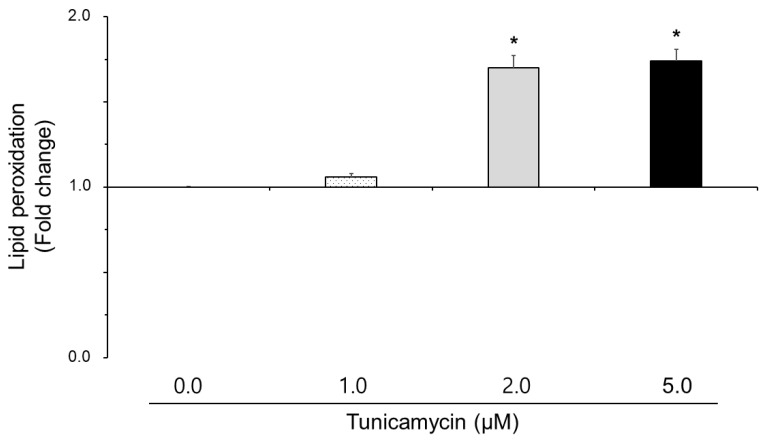
Effect of tunicamycin on lipid peroxidation in boar semen. Sperm were treated with 0.0, 1.0, 2.0, and 5.0 μM tunicamycin. Bars represent means ± standard error. Asterisks indicate significant differences (*, *p* < 0.05).

The effect of tunicamycin on the reactive oxygen species, nitric oxide, and lipid peroxidation is shown in Figure 1, Figure 2 and Figure 3. Reactive oxygen species and lipid peroxidation in the 2.0 μM and 5.0 μM tunicamycin treatment groups were significantly increased (*p* < 0.05). However, reactive oxygen species and lipid peroxidation were not significantly different in the 1.0 μM tunicamycin-treated group. And, nitric oxide in the tunicamycin-treated groups was not significantly different from that in the untreated groups.

## 4. Discussion

I investigated the relationship between sperm physiology function and oxidative stress in tunicamycin-treated sperm. The hypothesis of this experiment was that tunicamycin induces reactive oxygen species and lipid peroxidation. Thus, the cells are damaged by tumicamycin-regulated oxidative stress, including the motility and viability of sperm. In our study, exposure of boar sperm to tunicamycin resulted in decreased sperm motility and viability. These effects suggest that tunicamycin may influence sperm function, potentially through mechanisms involving oxidative stress. In humans, sperm exposed to oxidative stress exhibit activated autophagy, a cellular response to stress. Inhibition of the autophagic response leads to increased oxidative damage and cell death, underscoring the role of autophagy in maintaining sperm viability under stress conditions [17,18]. While direct studies on tunicamycin-induced oxidative stress in spermatozoa are scarce, existing research indicates that tunicamycin adversely affects sperm viability, mitochondrial activity, and motility. These effects may be mediated through oxidative stress pathways, highlighting the need for further research to elucidate the specific mechanisms involved.

Thus, we tested reactive oxygen species, nitric oxide, and lipid peroxidation of the sperm in tunicamycin-treated boar semen. In results, reactive oxygen species and lipid peroxidation in boar sperm were increased by tunicamycin, suggesting that tunicamycin induces reactive oxygen species, and activated reactive oxygen species enhance lipid peroxidation levels. Some papers reported that tunicamycin-induced reactive oxygen species generation leads to apoptosis through caspase-3 activation in leukemia cells [6]. The p38 mitogen-activated protein kinase (MAPK) signaling pathway plays a significant role in oxidative stress. In prostate cancer cells, tumicamycin-induced reactive oxygen species accumulation results in mitochondrial membrane potential loss and caspase-3 activation, culminating in cell death [19]. Tunicamycin-induced endoplasmic reticulum stress elevates reactive oxygen species production in chondrocytes, primarily through the nicotinamide adenine dinucleotide phosphate (NADPH) oxidase (NOX) system [20]. Tunicamycin-induced endoplasmic reticulum stress disrupts protein folding, leading to the accumulation of misfolded proteins. This stress activates the UPR, which in turn elevates reactive oxygen species production [21]. Modulating reactive oxygen species levels or enhancing antioxidant defenses could be potential approaches to mitigate tunicamycin-induced cellular damage in sperm. Although there are no studies on sperm, it is thought that tunicamycin induces reactive oxygen species and damages boar sperm, thereby reducing the cell viability.

Tunicamycin-induced endoplasmic reticulum stress disrupts the metabolism of sulfur-containing amino acids, crucial for glutathione synthesis. Reported by Kim, this disruption leads to decreased glutathione levels, impairing the liver’s antioxidant capacity and resulting in increased reactive oxygen species production [22]. Tunicamycin treatment decreased glutathione levels and potentiated cell death when exposed to tert-butyl hydroperoxide (t-BHP), a reactive oxygen species generator [22]. Glutathione synthesis is important for regulating lipid peroxidation. Reactive oxygen species initiate lipid peroxidation, resulting in elevated levels of malondialdehyde and 4-hydroxynonenal, which are markers of oxidative damage.

In sperm, endoplasmic reticulum stress can disrupt calcium homeostasis, leading to increased intracellular calcium levels. Elevated calcium levels activate phospholipases, enzymes that hydrolyze phospholipids in cellular membranes, generating arachidonic acid [23]. Arachidonic acid is a precursor for the synthesis of reactive oxygen species, which can initiate lipid peroxidation, resulting in sperm membrane damage and compromised motility. As seen from our results, tunicamycin-treated boar sperm increased reactive oxygen species and lipid peroxidation levels, suggesting that reactive oxygen species regulate lipid peroxidation conditions in boar sperm. In humans, oxidative stress has been shown to impair sperm motility by inducing lipid peroxidation, which affects membrane integrity and function [24]. This process highlights the vulnerability of spermatozoa to oxidative damage, including motility and fertilization capacity. In sperm, mitochondrial dysfunction is also associated with decreased adenosine triphosphate (ATP) production, essential for motility, and increased reactive oxygen species generation, contributing to lipid peroxidation and cellular damage [25]. Since lipid peroxidation in sperm membranes leads to compromised membrane integrity, altered fluidity, and impaired function, the damaged sperm membranes are less capable of undergoing the acrosome reaction, a prerequisite for fertilization. Overall, we suggest that lipid peroxidation products such as malondialdehyde and 4-hydroxynonenal can form adducts with deoxyribonucleic acid (DNA) and proteins, leading to mutations and functional impairments. Therefore, tunicamycin-induced lipid peroxidation may represent a significant threat to sperm quality and fertility. Also, tunicamycin-induced endoplasmic reticulum stress may lead to oxidative stress and lipid peroxidation in the boar sperm, primarily through the disruption of glutathione synthesis. Moreover, addressing this oxidative imbalance holds potential for therapeutic interventions in animal and human diseases associated with endoplasmic reticulum stress.

In our results, nitric oxide in boar sperm did not show any significant differences. Some papers indicated that tunicamycin in macrophage cells reduces lipopolysaccharide (LPS)-induced nitric oxide production by suppressing the expression of inducible nitric oxide synthase (iNOS). This effect occurs independently of endoplasmic reticulum stress and N-glycosylation pathways, suggesting a direct interaction between tunicamycin and the iNOS enzyme [26,27,28]. However, our tunicamycin study in boar sperm showed no changes. Thus, we suggest that tunicamycin influences nitric oxide production through multiple pathways, including the modulation of iNOS expression, endothelial NOS activity, and direct inhibition of nitric oxide synthase enzymes. These effects are cell-type dependent and can occur independently of endoplasmic reticulum stress and N-glycosylation inhibition. In the future, understanding these mechanisms provides insights into the broader pharmacological actions of tunicamycin and its potential therapeutic applications in other cells. Although tunicamycin showed oxidative stress effects, limitations of the study include the lack of an in vivo experiment and long-term toxicity assessment in the male physiology functions of pigs.

## 5. Conclusions

The results of our study were sufficient to demonstrate the potential of tunicamycin-regulated oxidative stress in boar sperm. Our current study may provide a novel role for tunicamycin on spermatozoa in pigs and other animals. Tunicamycin may be a powerful inducer of endoplasmic reticulum stress-mediated reactive oxygen species and lipid peroxidation in boar sperm. We suggest plausible functions of tunicamycin involving the generation of reactive oxygen species and lipid peroxidation in pigs. These roles can lead to lipid peroxidation and subsequent sperm dysfunction. Further research is warranted to elucidate the specific effects of tunicamycin on sperm lipid peroxidation and to assess the potential reproductive risks associated with tunicamycin exposure. Moreover, the reactive oxygen species-dependent signaling pathway is important in the mechanisms of apoptosis, viability, and sperm cell function.

## Figures and Tables

**Table 1:** Dose-dependent effect of tunicamycin on the motility and viability of sperm in boar semen.

	Tunicamycin (μM)				
	0.0	1.0	2.0	5.0	10.0
Motility	79.68 ± 1.88	79.82 ± 1.77	73.28 ± 0.99 *	71.48 ± 0.48 *	54.48 ± 1.35 *
Viability	65.08 ± 1.07	65.72 ± 0.77	55.44 ± 1.11 *	53.20 ± 0.78 *	40.00 ± 1.15 *

* Values in the same row with different superscripts are significantly different (*, *p* < 0.05), mean ± SEM.

## Data Availability

The original contributions presented in this study are included in the article. Further inquiries can be directed to the corresponding author.

## References

[1] Wu J., Chen S., Liu H., Zhang Z., Ni Z., Chen J., Yang Z., Nie Y., Fan D. (2018). Tunicamycin specifically aggravates ER stress and overcomes chemoresistance in multidrug-resistant gastric cancer cells by inhibiting N-glycosylation. J. Exp. Clin. Cancer Res..

[2] Yamamoto K., Ichikawa S. (2019). Tunicamycin: Chemical synthesis and biosynthesis. J. Antibiot..

[3] Lei Y., Wang S., Ren B., Wang J., Chen J., Lu J., Zhan S., Fu Y., Huang L., Tan J. (2017). CHOP favors endoplasmic reticulum stress-induced apoptosis in hepatocellular carcinoma cells via inhibition of autophagy. PLoS ONE.

[4] Hou H., Ge C., Sun H., Li H., Li J., Tian H. (2018). Tunicamycin inhibits cell proliferation and migration in hepatocellular carcinoma through suppression of CD44s and the ERK1/2 pathway. Cancer Sci..

[5] Anto E.M., Sruthi C.R., Krishnan L., Raghu K.G., Purushothaman J. (2023). Tangeretin alleviates Tunicamycin-induced endoplasmic reticulum stress and associated complications in skeletal muscle cells. Cell Stress Chaperones.

[6] Lim E.J., Heo J., Kim Y.H. (2015). Tunicamycin promotes apoptosis in leukemia cells through ROS generation and downregulation of survivin expression. Apoptosis.

[7] Kim S.H., Seo H., Kwon D., Yuk D.Y., Jung Y.S. (2022). Taurine Ameliorates Tunicamycin-Induced Liver Injury by Disrupting the Vicious Cycle between Oxidative Stress and Endoplasmic Reticulum Stress. Life.

[8] Chen F., Ge Z., Li N., Yu Z., Wu R., Zhao Y., He X., Cai G. (2022). TUDCA protects against tunicamycin-induced apoptosis of dorsal root ganglion neurons by suppressing activation of ER stress. Exp. Ther. Med..

[9] Shi J., Chen L., Wang X., Ma X. (2025). SIRT6 inhibits endoplasmic reticulum stress-mediated ferroptosis by activating Nrf2/HO-1 signaling to alleviate osteoarthritis. Inflamm. Res..

[10] Li C., Zhang B., Kim M., Liu H., Yang F., Chen K., Shi H. (2025). Atractylenolide partially alleviates tunicamycin-induced damage in porcine oocytes during in vitro maturation by reducing oxidative stress. Anim. Reprod. Sci..

[11] Yang L., Chen Z.H., Li J., Ding P.J., Wang Y. (2021). Effects of Escitalopram on Endoplasmic Reticulum Stress and Oxidative Stress Induced by Tunicamycin. Front. Neurosci..

[12] Lee S., Kim Y.M., Cheong H.T., Park C.K., Lee S.H. (2023). Effect of magnetized freezing extender on membrane damages, motility, and fertility of boar sperm following cryopreservation. Animals.

[13] Lee Y.S., Lee S., Lee S.H., Yang B.K., Park C.K. (2015). Effect of cholesterol-loaded cyclodextrin on sperm viability and acrosome reaction in boar semen cryopreservation. Anim. Reprod. Sci..

[14] Sung H.J., Jeong Y.J., Kim J., Jung E., Jun J.H. (2015). Soybean peptides induce apoptosis in HeLa cells by increasing oxidative stress. Biomed. Sci. Lett..

[15] Kojima H., Nakatsubo N., Kikuchi K., Kawajara S., Kirino Y., Nagoshi H., Hirata Y., Nagano T. (1998). Detection and imaging of nitric oxide with novel fluorescent indicators: Diaminofluoresceins. Anal. Chem..

[16] Singh M., Mollier R.T., Pongener N., Bordoloi L.J., Kumar R., Chaudhary J.K., Katiyar R., Khan M.H., Rajkhowa D.J., Mishra V.K. (2022). Linseed oil in boar’s diet during high temperature humidity index (THI) period improves sperm quality characteristics, antioxidant status and fatty acid composition of sperm under hot humid sub-tropical climate. Theriogenology.

[17] Uribe P., Merino J., Matus C.E., Schulz M., Zambrano F., Villegas J.V., Conejeros I., Taubert A., Hermosilla C., Sanchez R. (2022). Autophagy is activated in human spermatozoa subjected to oxidative stress and its inhibition impairs sperm quality and promotes cell death. Hum. Reprod..

[18] Sharma P., Kaushal N., Saleth L.R., Ghavami S., Dhingra S., Kaur P. (2023). Oxidative stress-induced apoptosis and autophagy: Balancing the contrary forces in spermatogenesis. Biochim. Biophys. Acta Mol. Basis. Dis..

[19] Guha P., Kaptan E., Gade P., Kalvakolanu D.V., Ahmed H. (2017). Tunicamycin induced endoplasmic reticulum stress promotes apoptosis of prostate cancer cells by activating mTORC1. Oncotarget.

[20] Kim Y.J., Han J., Han S. (2024). The interplay between endoplasmic reticulum stress and oxidative stress in chondrocyte catabolism. Sage J..

[21] Malhotra J.D., Kaufman R.J. (2007). The endoplasmic reticulum and the unfolded protein response. Semin. Cell Dev. Biol..

[22] Kim S.H., Kwon D.Y., Kwak J.H., Lee S., Lee Y.H., Yun J., Son T.G., Jung Y.S. (2018). Tunicamycin-Induced ER Stress is Accompanied with Oxidative Stress via Abrogation of Sulfur Amino Acids Metabolism in the Liver. Int. J. Mol. Sci..

[23] Beavers W.N., Monteith A.J., Amarnath V., Mernaugh R.L., Roberts L.J., Chazin W.J., Davies S.S., Skaar E.P. (2019). Arachidonic Acid Kills *Staphylococcus aureus* through a Lipid Peroxidation Mechanism. mBio.

[24] Agarwal A., Prabakaran S. (2005). Mechanism, measurement, and prevention of oxidative stress in male reproductive physiology. Indian J. Exp. Biol..

[25] Bansal A.K., Bilaspuri G.S. (2010). Impacts of oxidative stress and antioxidants on semen functions. Vet. Med. Int..

[26] Hosoi T., Noguchi J., Takakuwa M., Honda M., Okuma Y., Nomura Y., Ozawa K. (2014). Inhibition of inducible nitric oxide synthase and interleukin-1beta expression by tunicamycin in cultured glial cells exposed to lipopolysaccharide. Brain Res..

[27] Kucuksayan E., Konuk E.K., Demir N., Mutus B., Aslan M. (2014). Neutral sphingomyelinase inhibition decreases ER stress-mediated apoptosis and inducible nitric oxide synthase in retinal pigment epithelial cells. Free Radic. Biol. Med..

[28] Ohta S., Hattori Y., Nakanishi N., Sugimoto H., Kasai K. (2011). Differential modulation of immunostimulant-triggered NO production by endoplasmic reticulum stress inducers in vascular smooth muscle cells. J. Cardiovasc. Pharmacol..

